# Postdural Puncture Superior Sagittal Sinus Thrombosis in a Juvenile Case of Clinically Isolated Syndrome

**DOI:** 10.1155/2015/358164

**Published:** 2015-10-19

**Authors:** Miriam Michel, Edda Haberlandt, Matthias Baumann, Andreas Entenmann, Michaela Wagner, Kevin Rostasy

**Affiliations:** ^1^Department of Pediatrics, Medical University of Innsbruck, Anichstrasse 35, 6020 Innsbruck, Austria; ^2^Department of Neuroradiology, Medical University of Innsbruck, Anichstrasse 35, 6020 Innsbruck, Austria; ^3^Medical University of Witten/Herdecke, Department of Neuropediatrics, Dr.-Friedrich-Steiner Strasse 5, 45711 Datteln, Germany

## Abstract

*Background*. The causes of cerebral venous thrombosis (CVT) are manifold as is its clinical presentation. *Case*. We report the case of a CVT following lumbar puncture and intravenous glucocorticosteroid therapy in a female adolescent with a clinically isolated syndrome and risk factors for thrombosis. *Conclusion*. In adolescent patients with acute inflammatory disease undergoing lumbar puncture followed by intravenous high-dose glucocorticosteroid therapy, one should be aware of the elevated risk for thrombosis. A persistent headache with change in the headache pattern and loss of a postural component might be a sign for CVT, requiring emergency imaging of the brain.

## 1. Introduction

The causes of cerebral venous thrombosis (CVT) in children and adults are manifold as is its clinical presentation. In children, CVT is observed at any age with a higher incidence in neonates [1]. We report the case of a CVT following lumbar puncture and intravenous glucocorticosteroid therapy in a female adolescent with a clinically isolated syndrome and risk factors for thrombosis.

## 2. Clinical Presentation

A 17-year-old female with a prior medical history of idiopathic focal epilepsy with EEG continuous spike-and-wave during slow sleep (CSWSS), treated with sultiame for the last five years, and mild cognitive developmental delay presented with an unprovoked seizure during which she sustained a mild head trauma. A few days later her right arm and right leg felt “strange,” and she was unable to move her right body properly. Moreover, her parents noticed that she was slightly aggressive and that she was speaking less clearly. She was brought to the local hospital where an intracerebral haemorrhage was excluded by cranial computed tomography scan. She was referred to our clinic for further assessment and management. Neurological examination revealed a right-sided hemiplegia and a mild dysarthria. She further had a bilateral resting tremor worsening on finger-nose testing. Routine laboratory testing (complete blood cell count, electrolytes, protein, and liver and renal function parameters) was normal. There was no sign of systemic infection.

**Figure 1:**
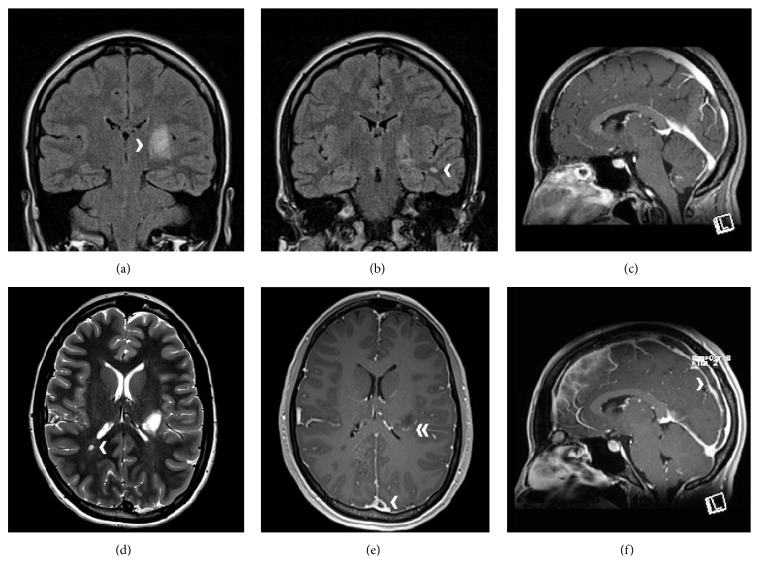
The magnetic resonance imaging of the patient reveals a large hyperintense lesion on FLAIR (a) and T2 (d) in the posterior limb of the left-sided internal capsule, probably extending to the thalamus. The other supratentorial lesions are much smaller (in the right occipital (d) and left temporal white matter ((b) FLAIR)). In the initial imaging the blood flow in the sagittal sinus was free ((c) gadolinium (Gd) enhanced T1w). 8 days later the white matter lesions were unchanged ((d) T2w, arrowhead pointing out a small lesion behind the occipital horn of the right ventricle) and some lesions were still Gd enhancing ((e) double arrowhead, Gd enhanced T1w). But now a thrombosis of the superior sagittal sinus can be seen ((e), (f) arrowheads, Gd enhanced T1w).

Cerebral magnetic resonance imaging (MRI) showed four supratentorial white matter lesions of high signal intensity in T2 and FLAIR enhancement in T1, the largest of them located in the posterior limb of the left-sided internal capsule and central region, and smaller lesions in the left temporal, right frontal, and right parietal region. Two lesions revealed contrast-medium enhancement (Figures 1(a), 1(b), and 1(d)). The venogram was completely normal without any evidence of poor flow, asymmetry, or stenoses of the sinuses.

A spinal MRI did not reveal any lesions. A lumbar puncture was conducted with a traumatic needle, revealing normal cerebral spinal fluid (CSF) pressure and normal total protein level. White cell count was elevated (14/*μ*L), and numerous additional CSF oligoclonal IgG bands were identified. Bacteriological findings were negative. Based on the aforementioned findings a diagnosis of a clinical isolated syndrome was made and the patient was treated with intravenous methylprednisolone pulse therapy (1 g daily) administered for five days [2, 3]. On the fourth day of steroid treatment, she developed severe and continuous frontotemporal headache, vertigo, and vomiting. The headache did not have any postural component and was unresponsive to intravenous analgesics. At first, these symptoms were assigned to the lumbar puncture and the steroid treatment. Because of persisting headache, a second cerebral MRI was performed showing a thrombosis of the superior sagittal sinus. There were no signs of venous congestion edema and no new white matter lesions (Figures 1(d)–1(f)). Anticoagulant treatment was started with low molecular weight heparin intravenously (2 mg/kgBW/d) and Phenprocoumon was introduced, shifting the international normalized ratio ((PT)-INR) to 2-3 [4]. In addition intravenous fluid therapy was administered. Under this therapy, headache and vomiting decreased and vanished within two days and the patient was discharged in good condition after ten days. Both the patient's and the family's thrombophilia history (deep vein thrombosis included) were negative. The patient's thrombophilia screening revealed normal values for the platelet count, antithrombin III, C and S protein, plasmatic homocysteine, fibrinogen, antinuclear antibodies, lupus anticoagulant, and cardiolipin antibodies. However, our patient carried a heterozygous mutation for factor II G20210A and a rather high lipoprotein A serum level, two factors known to increase the risk for thrombosis. Phenprocoumon therapy was planned for six months. Six weeks later the patient reported that she has had few minor headache episodes but no other symptoms. Her neurological examination was normal apart from the resting tremor of her hands. Ten weeks later the superior sagittal sinus was completely recanalised. The white matter lesion in the posterior limb of the left-sided internal capsule appeared smaller. None of the remaining lesions showed contrast-medium enhancement. There were no new lesions.

## 3. Discussion

CVT in paediatric patients is seen in various clinical settings such as infection, dehydration, trauma, renal failure, cancer, and haematological disorders [5]. Many children show additional prothrombotic risk factors [6]. In adults with multiple sclerosis CVT has been reported occurring shortly after a diagnostic lumbar puncture followed by intravenous high-dose glucocorticosteroid therapy [7–11], and only recently Presicci et al. reported a paediatric patient [12]. In our case the patient had a history of normal pregnancy, birth, neonatal period, and early childhood without any severe cases of trauma or dehydration [1]. She was not dehydrated during her hospital stay before the incident, nor was she obese, nor did she smoke. She did not take any medication (oral hormonal contraceptives included) other than sultiame which is not known for prothrombotic effects. However, our juvenile female patient was diagnosed with an acute inflammatory disease of the central nervous system and she underwent lumbar puncture followed by high-dose intravenous glucocorticosteroid treatment. Both factors, puncture and glucocorticosteroid treatment, raise the risk for the development of a thrombosis. By using a traumatic needle CSF leakage might appear, which might even be increased under the anti-inflammatory effect of the corticosteroid treatment possibly also inhibiting the reduction of the loss of dural tissue after the puncture. Thus, intracranial hypotension and a “rostrocaudal sagging” effect might be exerted on the intracranial contents. By a negative spinal-cranial pressure gradient, this hypotension might at the same time lead to venous endothelial traumatic damage from stretching of the cerebral vessels or to a stasis of the blood flow via venous dilatation, again possibly provoking the development of a central nervous thrombosis [9, 13]. Another important aspect is the fact that our patient carried a heterozygous mutation for factor II G20210A and a rather high lipoprotein A serum level, two factors known to increase the risk for thrombosis [14, 15]. The importance of hereditary and acquired prothrombotic disorders has been emphasized in recent series of paediatric CVT [1, 6, 12]. In our patient the epilepsy syndrome and the cephalalgia our patient suffered from might be regarded as an additional risk factor in the cascade of CVT [16, 17]. Interestingly, CSWSS is reported to possibly be related to prior sinus venous thrombosis, for example, in the straight sinus leading to thalamic compromise. Even if there is no imaging footprint this might at least subclinically have been the case in our patient. Thus, the episode after the minor head injury might have even been a recurrence [18].

Patient outcome depends on extent and location of cerebral parenchymal damage, haemoglobin, patient age, and the time interval between onset of symptoms and diagnosis and start of treatment. Permanent occlusion of parts of the intracranial venous drainage system, with or without formation of collaterals, may have an unfavourable impact on the developing brain [5]. Moreover, knowledge about prothrombotic factors for recurrence is important. Kenet et al. reported that the heterozygous factor II G20210A mutation, which was present in our patient, is a significant risk factor for recurrence of CVT, while a heterozygous factor V G1691A mutation or a raised lipoprotein(a) is not associated with an increased risk for recurrence [15].

## 4. Conclusion

In adolescent patients with acute inflammatory disease undergoing lumbar puncture followed by intravenous high-dose glucocorticosteroid therapy one should be aware of the elevated risk for thrombosis. We recommend at least asking for any additional risk factor for thrombosis in patients undergoing this type of diagnostic and treatment and if a patient complains of a persistent headache with change in the headache pattern and loss of a postural component, emergency imaging of the brain is mandatory; otherwise the diagnosis is likely to be missed.
